# Towards a standardised definition of full-time culture and its organisational facilitators and barriers among nursing care staff within Norwegian health services: a Delphi study protocol

**DOI:** 10.1136/bmjopen-2026-117970

**Published:** 2026-11-02

**Authors:** Kari Ingstad, Trude Anita Hartviksen, Marianne Mareliussen, Maria Strandås, Sinikka Lotvonen, Heidi Siira, Mojtaba Vaismoradi

**Affiliations:** 1 Faculty of Nursing and Health Sciences, Nord University, Levanger, Norway; 2 Center for Care Sciences, The Arctic University of Norway, Tromsø, Norway; 3 Vestvågøy Municipality, Vestvågøy, Norway; 4 Faculty of Nursing and Health Sciences, Nord University, Bodø, Norway; 5 GeroNursing Centre, University of Oulu, Oulu, Finland; 6 Faculty of Science and Health, Charles Sturt University, Orange, New South Wales, Australia

**Keywords:** Health Workforce, Health Services, Workplace

## Abstract

**Abstract:**

**Introduction:**

Part-time employment remains a persistent structural feature of Norway’s healthcare system, particularly in the provision of care during rotating work shifts. Despite longstanding political and institutional initiatives to promote full-time employment, a shared understanding of what constitutes a *full-time culture* and the organisational conditions that facilitate or hinder its development remains limited. This research aims to explore and establish expert consensus regarding the definition of a full-time work culture and the organisational facilitators of and barriers to developing and sustaining this culture among staff providing direct patient care and working in rotating work shifts within Norwegian healthcare services.

**Methods and analysis:**

A three-round, asynchronous online Delphi study will be conducted using the Welphi platform. Participants include 20 people recruited nationally who have relevant professional or experiential expertise in healthcare workforce organisation, staffing, management, policy, research and clinical practice. The same expert panel will participate across the Delphi rounds, with 20 participants expected in Round 1 and subsequent rounds subject to participant retention. Round 1 consists of open-ended questions exploring the meaning, characteristics, facilitators of and barriers to full-time culture. Their responses will be analysed thematically and synthesised into domains and statements which will form the basis for Round 2. In Rounds 2 and 3, participants will rate and re-rate the statements using a 5-point Likert scale, with anonymised group feedback between the rounds. They will also have opportunities to comment on and suggest modifications or additional statements. Consensus is defined a priori as ≥80% agreement (ratings of 4 or 5) and a median rating of ≥4.

**Ethics and dissemination:**

The study has been assessed by the Norwegian Agency for Shared Services in Education and Research (Sikt) (reference no. 710257). Participants will be asked to sign an electronic informed consent form before accessing the survey. Data management complies with the General Data Protection Regulation (GDPR) and institutional requirements. Findings will be disseminated through peer-reviewed publications, conference presentations and relevant professional networks and stakeholder-oriented reports.

**STRENGTHS AND LIMITATIONS OF THIS STUDY**

- This study seeks to address an underexplored area by developing a standardised definition of full-time culture for providing care to patients in rotating work shifts in the Norwegian healthcare system, an issue of high relevance given persistent healthcare workforce shortages.
- It seeks to capture diverse perspectives and avoid assumptions regarding the relative merits of full-time and part-time employment, leading to enhanced reflexivity.
- The Delphi approach will provide consensus-based knowledge about the potential benefits of full-time culture in an area with limited empirical evidence.
- As a consensus-based method, the study findings depend on the composition and perspectives of the expert panel and do not investigate empirical effectiveness.
- The findings from the Norwegian healthcare context should be transferred to settings with different labour market regulations or staffing models with caution and after further research.

## Introduction

 Demographic changes worldwide are driving a substantial increase in demand for long-term care services, particularly in home- and community-based sectors. The United Nations (UN) propose that the number of people aged 80 and older will become more than triple between 2015 and 2050, fuelling a sharp increase in long-term care needs, with most older adults preferring to receive care in their own homes.^1^ The number of users of formal home-based long-term care is expected to increase from approximately 192 000 in 2014 to around 330 000 by 2060, an increase of more than 70%.^2^ As older adults with long-term and complex health needs require more support in the community, understaffing has been identified as a major barrier to provide adequate and continuous support.^3^ Resource limitations are frequently reported in studies on home-based care because nursing staff shortages negatively affect the quality and safety of care.^4^

As part of the Nordic welfare model, Norwegian health services are founded on the principles of universal access and equal entitlement to care, irrespective of income, social status or place of residence. Services are accordingly organised and designed to ensure that as many people as possible can remain active in the workforce. Health services are primarily publicly financed through taxation, supplemented by patient co-payments.^5^ Only about 5–10% of services are delivered by private sectors, and the majority of these services are also purchased by the public sector.^6^ While the Norwegian healthcare system is designed to provide universal and equitable access to care, its ability to fulfil these ambitions depends on a stable and sustainable workforce. In this respect, staffing patterns within the health and care sector represent an important contextual factor.

In Norway, part-time employment is widespread: 46% of women and 25% of men work part-time.^7^ These rates are particularly high in healthcare services that operate on a 24/7 basis and rely on rostered shift work, including day, evening, night and weekend shifts. Across Europe, 28% of employed women aged 15–64 work part-time compared with only 8% of men, with women representing the highest share across all occupational categories.^8^ In both municipal healthcare services and hospitals, patient care is largely delivered by staff working on rotating work shifts outside standard daytime hours. In the present study, it includes registered nurses, licensed practical nurses, healthcare workers, nursing assistants and care staff without formal health or social care education. In contrast, physicians are rarely employed involuntarily part-time^9^ and are not a focus of the present study. Accordingly, part-time employment is a persistent structural feature. It has been estimated that 43% and 72% of nurses and auxiliary nursing staff work part-time.^9^ In the municipal healthcare system, only 47% of nursing positions are full-time, indicating a consistently high prevalence of part-time employment among nurses in this sector in Norway.^10^ The widespread use of part-time employment in Norwegian healthcare has been partly attributed to working-time reforms introduced in 1987. The transition from working every second weekend to every third weekend has increased staffing requirements, leading to a greater reliance on part-time positions to ensure adequate weekend coverage.^11^ Part-time employment has also been embedded in staffing models and working-time arrangements; for example, the average weekly working time for women in Norway is 32.4 hours, compared with 34.3 hours in the EU, 36.7 hours in Sweden and 33.8 hours in Finland (12, p5).^12^

The consequences of the high prevalence of part-time employment are multiple and complex. As a paradox, despite ongoing shortages of healthcare personnel, those staff providing direct patient care work in rotating work shifts and mostly in involuntary part-time positions. In Norway, approximately 68 000 employees are involuntarily employed on a part-time basis, representing 14.9% of all employees working part-time.^13^ Approximately one in four part-time employees report that they would prefer a full-time position.^14^ Healthcare assistants and other staff are among the occupational groups with the highest prevalence of involuntary part-time work, and the health and care sector accounts for the largest share of involuntary part-time employment.^15^

Increasing employment fractions among shift-working care staff represents a major opportunity to address workforce shortages in both municipal and hospital settings. Employing all nurses and healthcare assistants in positions of at least 70% would correspond to approximately 2700 additional full-time equivalents by 2025. A transition to full-time employment for all care staff would yield an estimated 43 300 full-time employees.^9^ There is also considerable potential to increase employment fractions among patient-facing staff without formal health or social care education, many of whom work in fragmented shifts across multiple units or for various employers.

Increasing the proportion of full-time employment can yield benefits beyond simply expanding the available workforce. Part-time employment is associated with lower work involvement, affective commitment and work engagement, fewer job resources and particularly when part-time employment is involuntary, lower professional self-confidence and a sense of disconnection from the workplace.^16–18^ Part-time employment can also affect continuity and consistency of care, with an impact potentially depending on how working time and shift schedules are organised, but evidence regarding patient experiences and professional collaboration is mixed.^18–20^ Increasing employment fractions, particularly by reducing involuntary part-time employment, can support not only workforce capacity but also employee engagement, professional development, continuity of care and the long-term sustainability of healthcare services.

The promotion of full-time positions and a *full-time culture* has been on the Norwegian political agenda for more than 30 years. Despite several national initiatives, social partners have yet to reach solutions that produce significant results. Although the share of full-time employees has increased since 2014,^15 21^ the healthcare sector has not achieved a genuine full-time culture, indicating considerable room for further improvement.^22^ Developing a full-time culture has long been an explicit goal for both the government and social partners. Social partners such as (The Norwegian Association of Local and Regional Authorities (KS), Fagforbundet, Norwegian Nurses Organisation (NSF), Delta and The Norwegian Union of Social Educators and Social Workers (FO)) have signed the *Heltidserklæring*, a declaration of intent to promote full-time work, on four occasions: in 2013, 2015, 2018 and 2021.^23^ The declaration encourages municipalities to create larger positions and foster an organisational culture that supports full-time employment.

International literature suggests that a key obstacle to establishing safe staffing levels is the systematic underestimation of nursing workforce needs, combined with an overestimation of substitution possibilities, leading to insufficient baseline staffing and persistent nurse shortages.^24^ Inappropriate staffing patterns, characterised by nurse shortages and high levels of part-time employment in healthcare, are linked to fragmented shifts, recruitment challenges and workforce instability. In addition, patients’ evaluations of hospital care are strongly associated with the prevalence of missed nursing care, which in turn is linked to inadequate nurse staffing and an unfavourable hospital work environment.^25^ Improvements in nurses’ job satisfaction and perceptions of the safety climate are also influenced by staffing adequacy and the availability of sufficient human resources (HR).^26^ Higher nurse staffing is likely to foster better patient outcomes and prevent patient mortality.^27 28^ Improving staffing by reducing the patient-to-nurse ratio by one is associated with reductions in mortality (OR: 0.93, 95% CI 0.86 to 0.99, p=0.045), readmissions (OR: 0.93, 95% CI 0.89 to 0.97, p<0.0001) and length of stay (IRR: 0.97, 95% CI 0.94 to 0.99, p=0.035). The savings generated through fewer readmissions and shorter hospital stays exceed two times the cost of increased nurse staffing.^29^ Therefore, strengthening appropriate staffing patterns and full-time work are considered key strategies for ensuring sustainable staffing, improving continuity of care and supporting the quality and safety of care.

The high prevalence of part-time employment, the continued demand for larger employment fractions and persistent challenges in establishing a full-time culture indicate that increasing full-time employment is not simply a matter of creating more full-time positions. Rather, it requires changes in organisational practices, staffing models, working-time arrangements and shared perceptions of what constitutes feasible and desirable employment. Despite longstanding policy efforts, there is still limited consensus on what a full-time culture should entail in practice, the organisational conditions required to support its development and the factors that facilitate or hinder its adoption among nursing staff providing direct patient care in working rotating shifts. This gap of knowledge as the rationale for conducting the present study led to devising a Delphi approach to develop expert consensus on the meaning of full-time culture and identify key factors that can support or hinder its development in Norwegian healthcare services.

### Conceptual basis

The organisational challenges suggest that developing a full-time culture involves more than increasing employment fractions. The concept of organisational culture provides a useful framework for understanding how perspectives, norms and practices related to full-time work develop and persist. Schein^30^ defined organisational culture as a set of shared beliefs and assumptions formed through external and internal integration. Similarly, Akhavan *et al*^31^ described it as the collective norms, values and behaviours that shape how members of an organisation think and act. Full-time culture can be understood as part of a broader organisational culture, reflecting shared assumptions about what is normal, desirable and possible regarding working time and staffing practices.

However, there is neither an agreed-upon definition of a full-time culture in this context nor a clear consensus regarding the organisational conditions, facilitators of and barriers to developing and sustaining it. This lack of conceptual clarity and consensus limits our ability to design effective measures for establishing a full-time culture in healthcare systems. Therefore, a clearer conceptual understanding of what a full-time culture entails and which factors influence its development in practice is needed. This study uses a Delphi technique to assess the level of consensus among a panel of experts regarding the definition, facilitators of and barriers to developing and sustaining a full-time culture in Norwegian healthcare. The study will also identify which statements reach the predefined level of consensus and describe the degree of agreement for statements that do not reach this threshold.

### Study aim

The primary aim of this Delphi study is to explore and establish expert consensus regarding the definition of a full-time work culture and the organisational facilitators of and barriers to developing and sustaining this culture among staff providing direct patient care and working in rotating work shifts within Norwegian healthcare services.

### Research questions

**RQ1:** How do experts define *full-time culture* for staff providing direct patient care and working in rotating work shifts within Norwegian healthcare services?

**RQ2:** What are the facilitators of and barriers to developing full-time culture in this context?

## Methods and analysis

### Study design

This study will use a Delphi technique to obtain consensus on a particular topic through multiple rounds of questionnaires administered to experts in the field, who remain anonymous and receive feedback between each round.^32^ This method is widely used in health sciences to achieve expert consensus and establish shared definitions.^33–36^

#### Study overview

The Delphi technique will be used electronically and asynchronously by the research team via Welphi.com (www.welphi.com) as an online survey platform designed for conducting Delphi research and analysing the collected data.

The Delphi study will begin with an exploratory open-ended Round 1 in which all participating experts will respond to questions addressing the meaning, characteristics, facilitators of and barriers to a full-time culture for staff providing direct patient care and working in rotating work shifts within Norwegian healthcare. Responses will be analysed thematically and synthesised into preliminary domains and statements. In subsequent Delphi rounds, the same expert panel will anonymously rate and re-rate these statements using a 5-point Likert scale, with anonymised feedback provided between rounds. Participants will have opportunities to comment on, refine or suggest additional statements throughout the consensus process. Consensus will be defined a priori as ≥80% agreement (ratings 4 or 5) and a median rating of ≥4.

Experts will be invited via email with a link to the online survey, which will include the participant information sheet, consent form and a sociodemographic data form capturing the name, job title, organisation and relevant experience with full-time work culture.

Upon obtaining informed consent, three Delphi rounds will be conducted. In Round 1, experts will respond to a series of open-ended questions exploring their perspectives on the characteristics, facilitators of and barriers to full-time work culture. Responses from Round 1 will be analysed thematically and synthesised into domains and statements. These domains and statements will form the basis for Round 2. In Round 2, experts will rate these statements using a 5-point Likert scale (1=strongly disagree, 2=disagree, 3=neutral, 4=agree, 5=strongly agree). Open-text fields will allow participants to provide additional comments and suggest modifications to the statements. Round 3 will provide participants with feedback from the previous round and an opportunity to re-rate the statements and provide further comments to refine consensus.

Each round will take approximately 1 month to complete, and participants will have 2 weeks to respond. Reminder emails will be sent 1 week, 2 days and 1 day before the close of each round, as well as on the closing day. After 2 weeks, non-responders will receive an email offering an additional 3 days to complete the survey. If they do not respond within this period, they will be excluded from the study. The research team will use the remaining 2 weeks of each round to complete data analysis and develop the subsequent survey round based on participants’ responses. An overview of the Delphi process and study rounds is presented in figure 1. In addition, table 1 presents the planned timeline for recruitment, Delphi rounds, data analysis and reporting.

**Figure 1:**
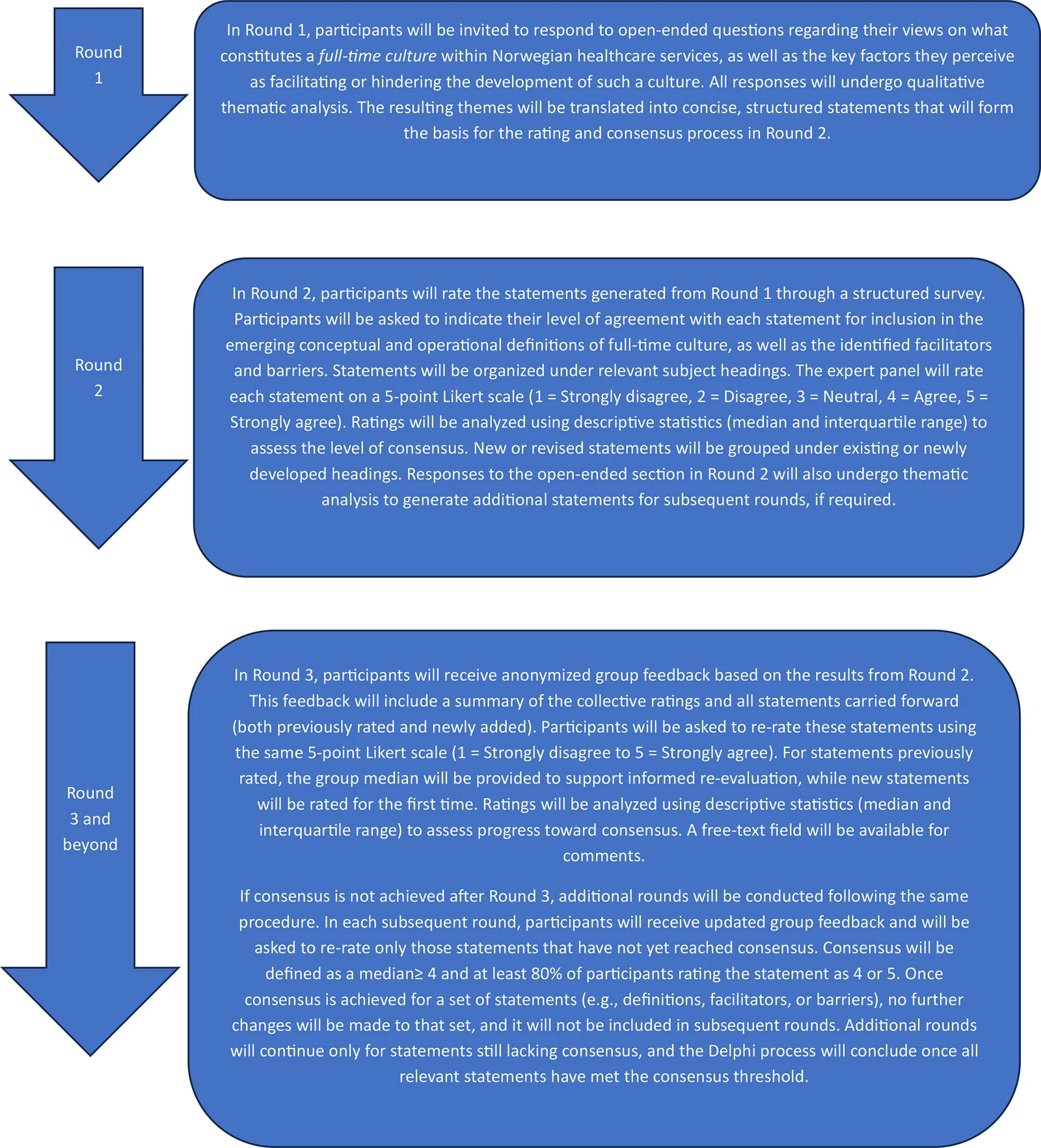
Overview of the Delphi process and study rounds.

**Table 1:** Planned timeline for recruitment, Delphi rounds, data analysis and reporting

Phase	Activities	Timeline
Round 1	Recruitment and panel confirmation	Months 1–2
Analysis	Data analysis using qualitative and quantitative methods	Months 2–3
Round 2	Restructuring, rating and feedback	Months 3–4
Round 3	Restructuring, rating and feedback, consensus survey	Months 4–5
Reporting	Final analysis, manuscript preparation	Months 5–7

Prior to data collection, the online questionnaire and survey procedures on Welphi will be pilot tested by two researchers and three healthcare leaders who have relevant experience related to the study topic. The pilot testing aims to assess the functionality, readability and usability of the survey platform and study materials. Responses from the participants in the pilot testing will not be included in subsequent analyses.

Data collection is planned to take place between January and July 2027.

#### Participants

There are no standardised guidelines or consensus in the literature regarding the definition of an ‘expert’ in Delphi studies.^32 37^ Niederberger and Spranger^33^ suggest that an expert can be defined either through formal expertise or lived experience. Aligning with this perspective, a diverse group of experts will be defined based on their relevant knowledge, professional experience and capacity to provide informed input on a full-time healthcare culture. The expert panel will aim for a balanced representation across different healthcare disciplines, educational levels and geographical regions.

To ensure a comprehensive and diverse range of perspectives, the expert panel will be representatives from the following groups:

1. *Researchers and academic experts* engaged in research on staffing models and full-time culture in healthcare, affiliated with institutions such as SINTEF, FAFO, OsloMet, University of Stavanger and VID Specialised University.
2. *Healthcare management and leadership*, such as municipal and regional healthcare managers, HR directors and workforce planners, with direct experiences in initiatives related to full-time culture.
3. *Employee representatives and labour market actors*, which will include representatives from the Norwegian Nurses Organisation (NSF), KS, Spekter, Delta and Fagforbundet.
4. *Policy and government representatives*, including officials or advisers from the Ministry of Health and Care Services and municipal policymakers, involved in workforce planning and development at the regional and national levels.
5. *Professional and practitioner representatives* with practical experiences and insights into part-time and full-time work culture within Norwegian healthcare services.

This composition is intended to capture multiple dimensions of expertise, ensuring that the Delphi process reflects both empirical knowledge and experiential understanding of the facilitators of and barriers to establishing full-time culture in Norwegian healthcare services.

Patient and family representatives will not be included in the Delphi panel. As the primary aim of this research is to develop consensus on the definition of full-time culture and the organisational factors influencing its development, participants will be chosen based on their expertise and experience in workforce organisation, staffing, management, policy, research or clinical practice within the healthcare sector. Although patients and family members may provide valuable perspectives on care experiences, the focus of this study is on workforce organisation, staffing models and employment structures within healthcare services. Therefore, participation in the Delphi panel is limited to individuals with relevant professional expertise and experience in workforce planning, management, policy, research or clinical practice.

There is no consensus in the literature regarding the optimal size of expert panels for Delphi studies.^32 33^ The size of a Delphi panel is determined by group dynamics to achieve expert consensus, rather than statistical power. Accordingly, the literature recommends 10–18 experts.^38^

Given the national scope of this study and its focus on Norwegian healthcare, 20 participants will be recruited for inclusion in the expert panel in this research.

#### Recruitment

Norwegian experts will be recruited to ensure representation from key stakeholder groups relevant to full-time culture in healthcare. The research team compiles a list of potential participants considering inclusion and exclusion criteria listed in table 2.

**Table 2:** List of inclusion and exclusion criteria for the selection of participants

Inclusion criteria	Exclusion criteria
Researchers and academic members who have published articles on full-time culture or related workforce topics in Norwegian healthcare services	Individuals without relevant experience or expertise related to full-time culture, staffing, workforce planning or employment arrangements in healthcare
Healthcare managers and leaders, including municipal and regional managers, HR directors and workforce planners with experience in implementing, managing or supporting full-time culture initiatives in municipalities and hospitals	Individuals participating only in the pilot testing
Employee representatives and labour market actors, such as trade unions and employer organisations such as Norwegian Nurses Organisation (NSF), Fagforbundet, (the Norwegian Association of Local and Regional Authorities (KS), Spekter, Delta, who can nominate internal experts	Patients, service users and relatives, as they are not expected to have the specific organisational and workforce-related expertise required for the Delphi process
Policy and government representatives, including officials and advisers from the Ministry of Health and Care Services and municipal policymakers, involved in workforce planning	Individuals unable or unwilling to participate in subsequent Delphi rounds
Practitioner representatives with practical experiences and insights into part-time and full-time work practices and rotating work shifts in healthcare services	

HR, human resource.

Potential experts will be identified through publications, organisational records and recommendations from professional associations. Snowball sampling will also be used to identify probable participants.

To ensure balanced representation, registration will be monitored using a matrix to track diversity across professional backgrounds, organisational affiliation and experience with full-time culture initiatives. Once the target panel size has been reached, the first Delphi round will be commenced, with participants notified via email 1 week in advance.

#### Consensus

There are no standardised guidelines or universal agreements in the literature regarding what constitutes consensus in Delphi studies.^32 33^ Consensus has been defined as a predetermined percentage of participants in agreement, typically ranging from 51% to 100%.^32^ In this research, consensus has been defined a priori as ≥80% agreement (ratings 4 or 5) and a median rating of ≥4. This threshold is chosen based on previous Delphi studies in health sciences, where consensus is commonly defined as ≥70%, ≥75% or ≥80%,^39 40^ with ≥80% representing the most stringent level.

### Data analysis

Quantitative data will be analysed descriptively using the median and IQR, alongside qualitative thematic analysis of open-ended responses. These analyses will reflect both consensus in terms of level of agreement and stability as consistency across rounds. Items for which at least 80% of participants rate their agreement as agree and strongly agree, corresponding to 4 and 5 on the Likert scale, will be included in the preliminary conceptual and operational definitions of full-time culture. Qualitative thematic analysis of participants’ comments will help refine these definitions.

Given the anticipated expert panel size of 20 participants and the diversity of professional profiles, this study is not designed or powered to support meaningful comparisons between stakeholder subgroups. The primary objective is to establish overall expert consensus across the panel. Participant characteristics will nevertheless be described, and patterns of agreement across stakeholder groups will be explored descriptively where the number of participants permits, without interpreting such differences as statistically generalisable subgroup effects.

### Quality and transparency

This study will be conducted and reported in accordance with the Conducting and Reporting Delphi Studies guidelines.^41^ The Delphi process will be structured to ensure transparency, methodological rigour and traceability of responses across rounds. Data will be organised to allow accurate follow-up of individual responses over successive Delphi rounds, enabling systematic feedback while maintaining participant confidentiality.

### Ethics and data management

This study will be conducted in accordance with established ethical guidelines for research involving human participants. Informed consent will be obtained from all participants prior to data collection. Data protection approval has been obtained from Sikt (reference no. 710257).

Data management will be conducted in accordance with Nord University’s data management protocol and the General Data Protection Regulation. A data processing agreement has been established between data controller at Nord University and data processor on Welphi.com. All data, including questionnaires, ratings and expert comments, will be stored securely on university-approved servers with access restricted to the research team. Regular backup will be performed to prevent data loss. On completion of the study, the data will be archived in accordance with institutional guidelines, with due consideration of anonymity and confidentiality.

#### Expected outcomes

A standardised definition of *full-time culture* in Norwegian healthcare services will be developed, along with the identification of facilitators of and barriers to establishing and sustaining a full-time culture within the Norwegian healthcare services. The findings are expected to inform organisational strategies and policy development aimed at strengthening workforce stability and improving continuity and quality of care.

## Discussion

Across Europe, including Norway, part-time work remains a structural feature of the healthcare workforce. Despite longstanding political and institutional initiatives, the proportion of part-time employees, particularly among nurses and healthcare assistants, remains high. This persistent pattern limits workforce flexibility and contributes to staffing shortages, fragmented shifts, reduced continuity of care and an inability to meet the growing demand for long-term community healthcare services. The present Delphi study aims to contribute to a shared understanding of *full-time culture* by bringing together knowledge and perspectives from experts with different professional and organisational backgrounds. By identifying areas of consensus as well as facilitators and barriers perceived across different expert profiles, this study can support knowledge sharing and inform the development of context-sensitive strategies for strengthening full-time culture in Norwegian healthcare. Differences in perspectives between stakeholder groups can also help identify areas where targeted approaches or further dialogue are needed. In addition, the findings can highlight gaps in current knowledge and provide directions for future research on how full-time culture can be developed and sustained in different healthcare contexts.

This study views full-time culture as an integral part of the broader organisational culture. Shared values and assumptions shape everyday practices and influence how staffing and employment arrangements are implemented. Given the importance of organisational culture for the well-being of both patients and healthcare professionals,^42^ understanding these underlying beliefs can help managers and policymakers move beyond structural and financial measures toward sustainable cultural and behavioural change.

Our research implications extend beyond workforce planning. A more stable and full-time workforce has been shown to improve job satisfaction, reduce turnover and enhance patient outcomes.^25 27^ By identifying cultural, organisational and systemic barriers to full-time employment, this study can inform targeted initiatives that not only address healthcare personnel shortages but also promote professional development, predictability and patient safety.

Importantly, the purpose of this study is not to assume that full-time employment is inherently preferable to part-time employment. Rather, it aims to explore expert perspectives on the opportunities, barriers and potential consequences associated with developing a stronger full-time work culture. Recognising that both full-time and part-time employment can offer advantages and disadvantages for employees, organisations and service users, the study seeks to provide a nuanced understanding of the cultural, organisational and systemic factors that shape staffing patterns in healthcare.

While the Delphi technique provides a rigorous and structured approach to consensus building, it also depends on the diversity and representativeness of the expert panel. Including stakeholders from multiple levels, encompassing policy, management and practice, can strengthen the validity and relevance of the results. Future research should explore how the agreed definitions and factors can be operationalised and tested in practice through implementation or intervention studies.

Nevertheless, by strengthening conceptual clarity and establishing expert consensus, this study is expected to provide a structured foundation for identifying the key facilitators of and barriers to developing a full-time culture among staff providing direct patient care and working in rotating work shifts within Norwegian healthcare services. The study findings can inform the development of practical strategies, policies and organisational initiatives aimed at addressing barriers to and supporting a sustainable full-time culture in short-term and long-term healthcare services.

## References

[1] United Nations (2016). Growing need for long-term care: assumptions and realities [briefing paper]. https://www.un.org/development/desa/ageing/news/2016/09/briefing-paper-growing-need-for-long-term-care-assumptions-and-realities/.

[2] Statistics Norway (2016). Demand for labour and residential care in the long term care sector towards 2060. https://www.ssb.no/en/helse/artikler-og-publikasjoner/behovet-for-arbeidskraft-og-omsorgsboliger-i-pleie-og-omsorgssektoren-mot-2060.

[3] Ploeg J, Yous M-L, Fraser K (2019). Healthcare providers’ experiences in supporting community-living older adults to manage multiple chronic conditions: a qualitative study. BMC Geriatr.

[4] Sørstrøm AK, Kymre IG, Ludvigsen MS (2024). Nursing care to patients who have the home as the preferred place of death: a scoping review. BMC Health Serv Res.

[5] Sandvin JT, Vike H, Anvik CH, Sandvin JT, Breimo JP (2020). Velferdstjenestenes vilkår. Nasjonal politikk og lokale erfaringer [Terms of welfare services: national politics and local experiences].

[6] Jakobsen EW, Lind LH, Halvorsen CA (2021). Ideelle og kommersielle aktører i helse- og omsorgssektoren.

[7] Røv V (2024). Deltidsjobber utgjør nesten en tredel av alle jobber [part-time jobs account for almost a third of all jobs]. https://www.ssb.no/arbeid-og-lonn/sysselsetting/statistikk/antall-arbeidsforhold-og-lonn/artikler/deltidsjobber-utgjor-nesten-en-tredel-av-alle-jobber.

[8] Eurostat Share of women working part-time higher than men 2023. https://ec.europa.eu/eurostat/web/products-eurostat-news/w/edn-20230303-1.

[9] Jia Z, Kornstad T, Stølen NM (2023). Arbeidsmarkedet for helsepersonell fram mot 2040 [the labour market for health personnel towards 2040].

[10] Norsk Sykepleierforbund (NSF) Statistics. https://www.nsf.no/lonn-og-tariff/statistikk.

[11] Moland LE (2013). Heltid-deltid – en kunnskapsstatus [full-time and part-time employment: a review of the evidence].

[12] NOU 2025: 5 kvinners arbeidshelse: kunnskap og tiltak [women’s occupational health: knowledge and measures].

[13] Statistics Norway (SSB) (2025). Labour force survey. https://www.ssb.no/arbeid-og-lonn/sysselsetting/statistikk/arbeidskraftundersokelsen.

[14] Nielsen RA, Bråthen K, Moland LE (2026). Deltidsansettelser og bærekraftig bemanning [part-time employment and sustainable staffing]. https://www.fafo.no/images/pub/20979_Deltidsansettelser_og_bærekraftig_bemanning_med_KS-logo.pdf.

[15] Bråthen K (2024). Fafos forskning og kunnskapsutvikling på heltid-deltidsfeltet de siste 15 årene [fafo’s research and knowledge development on full-time and part-time work over the last 15 years]. https://www.regjeringen.no/contentassets/3851f71301cf4747875a444d10b7b379/no/sved/vedl05.pdf.

[16] Ingstad K (2025). Heltidskultur.

[17] J. Burke R, L. Dolan S, Fiksenbaum L (2014). Part-time versus full-time work: an empirical evidence-based case of nurses in Spain. Evidence-Based HRM.

[18] Jelodar ZK, Saghafi F, Zackery A (2023). Picture of the employment status of nurses in the world: Scoping review on pros and cons. J Educ Health Promot.

[19] Jamieson LN, Williams LM, Lauder W (2008). The “realities” of part-time nursing: a grounded theory study. J Nurs Manag.

[20] Magerøy MR, Wiig S (2023). The effect of full-time culture on quality and safety of care – a literature review. *IJHG*.

[21] Regjeringen (2025). Økt bruk av heltidsstillinger i norske kommuner [increased use of full-time positions in norwegian municipalities]. https://www.regjeringen.no/no/aktuelt/okt-bruk-av-heltidsstillinger-i-norske-kommuner/id3125552.

[22] Moland LE, Tofteng M, Bråthen K (2023). Økonomiske kostnader og gevinster ved å utvikle heltidskultur i kommunale pleie- og omsorgstjenester [what does full-time actually cost? economic costs and benefits of developing a full-time culture in municipal nursing and care services].

[23] Sentralforbund K, Kommunenes Sentralforbund (KS) (2021). Det store heltidsvalget 2021 – signert heltidserklæring [the big full-time choice 2021 – signed full-time declaration]. https://www.ks.no/fagomrader/arbeidsgiverpolitikk/heltid/det-store-heltidsvalget-2021--signert-heltidserklaring.

[24] Ball JE, Griffiths P (2022). Consensus Development Project (CDP): An overview of staffing for safe and effective nursing care. Nurs Open.

[25] Aiken LH, Sloane DM, Ball J (2018). Patient satisfaction with hospital care and nurses in England: an observational study. BMJ Open.

[26] Dutra CKDR, Guirardello E de B (2021). Nurse work environment and its impact on reasons for missed care, safety climate, and job satisfaction: A cross-sectional study. J Adv Nurs.

[27] Dall’Ora C, Saville C, Rubbo B (2022). Nurse staffing levels and patient outcomes: A systematic review of longitudinal studies. Int J Nurs Stud.

[28] Lasater KB, Aiken LH, Sloane D (2021). Patient outcomes and cost savings associated with hospital safe nurse staffing legislation: an observational study. BMJ Open.

[29] McHugh MD, Aiken LH, Sloane DM (2021). Effects of nurse-to-patient ratio legislation on nurse staffing and patient mortality, readmissions, and length of stay: a prospective study in a panel of hospitals. Lancet.

[30] Schein EH (1992). Organizational culture and leadership.

[31] Akhavan P, Ebrahim Sanjaghi M, Rezaeenour J (2014). Examining the relationships between organizational culture, knowledge management and environmental responsiveness capability. *VINE*.

[32] Chalmers J, Armour M, Liamputtong P (2019). Handbook of research methods in health social sciences.

[33] Niederberger M, Spranger J (2020). Delphi Technique in Health Sciences: A Map. Front Public Health.

[34] Metzelthin SF, Rostgaard T, Parsons M (2022). Development of an internationally accepted definition of reablement: a Delphi study. Ageing Soc.

[35] Allen J, Brenner M, Hauer J (2020). Severe Neurological Impairment: A delphi consensus-based definition. Eur J Paediatr Neurol.

[36] Campbell KA, Wood JN, Lindberg DM (2021). A standardized definition of near-fatal child maltreatment: Results of a multidisciplinary Delphi process. Child Abuse Negl.

[37] Puig A, Adams C (2018). Delphi technique.

[38] Okoli C, Pawlowski SD (2004). The Delphi method as a research tool: an example, design considerations and applications. *Information & Management*.

[39] Diamond IR, Grant RC, Feldman BM (2014). Defining consensus: a systematic review recommends methodologic criteria for reporting of Delphi studies. J Clin Epidemiol.

[40] Muhl C, Mulligan K, Bayoumi I (2023). Establishing Internationally Accepted Conceptual and Operational Definitions of Social Prescribing Through Expert Consensus: A Delphi Study Protocol. Int J Integr Care.

[41] Jünger S, Payne SA, Brine J (2017). Guidance on Conducting and REporting DElphi Studies (CREDES) in palliative care: Recommendations based on a methodological systematic review. Palliat Med.

[42] Efremov L, Dimitrievska V, Kallach L (2025). Digitalization and management innovation iii.

