# Supplementary material for: Towards a standardised definition of full-time culture and its organisational facilitators and barriers among nursing care staff within Norwegian health services: a Delphi study protocol
Source: BMJ Open. 2026 Nov 2;16(11):e117970. doi: 10.1136/bmjopen-2026-117970 (PMC13735351; doi:10.1136/bmjopen-2026-117970)
Supplement: Supplementary file 1 [file bmjopen-2026-117970.reviewer_comments.pdf]

PEER REVIEW HISTORY

BMJ Open publishes all reviews undertaken for accepted manuscripts. Reviewers are asked to complete a checklist review form and are provided with free text boxes to elaborate on their assessment. These free text comments are reproduced below.

ARTICLE DETAILS

Title (Provisional)

Towards a Standardised Definition of Full-Time Culture and Its Organisational Facilitators and Barriers Among Nursing Care Staff Within Norwegian Health services: A Delphi Study Protocol

Authors

Ingstad, Kari; Hartviksen, Trude Anita Anita; Mareliussen, Marianne; Strandås, Maria; Lotvonen, Sinikka; Siira, Heidi; Vaismoradi, Mojtaba

---

VERSION 1 - REVIEW

**Reviewer**	**1**
**Name**	**Park, Jin Hee**
**Affiliation**	**Changshin University, Nursing**
**Date**	**13-May-2026**
**COI**	**None**

Thank you for the opportunity to review your manuscript. The topic is relevant and timely; however, several areas require further clarification and strengthening to enhance the rigor and clarity of the paper.

First, in the section addressing the background and necessity of the study, the description of healthcare workforce data in Norway is currently too brief. I recommend expanding this part by incorporating recent statistics related to the "full-time work culture" among healthcare professionals in Norway. In particular, please clarify the extent to which full-time positions are lacking, why such shortages persist, and what structural or organizational factors contribute to this issue. Additionally, it would be important to explicitly discuss the consequences of this shortage, such as its impact on continuity of care, staff well-being, quality of services, and organizational efficiency. Providing this context will help readers better understand the significance and urgency of the research problem.

Second, the purpose of the study would benefit from clearer alignment with the identified gaps. Specifically, please articulate more explicitly how the lack of a full-time work culture and related workforce challenges motivated the present study. Making this connection more

explicit will improve the logical flow of the manuscript and help readers better appreciate the contribution of your research.

Finally, in the Discussion section, some of the cited references appear outdated. For example, references [17] (1992) and [18] (2014) are more than a decade old. Given the rapid changes in healthcare systems and organizational culture, these sources may not adequately reflect current hospital work environments. I strongly recommend replacing or supplementing these references with more recent literature that captures contemporary organizational culture in healthcare settings. This will strengthen the validity and relevance of your discussion.

I hope these comments are helpful in revising your manuscript.

**Reviewer**	**2**
**Name**	**An , Delian**
**Affiliation**	**Third Affiliated Hospital of Sun Yat-Sen University**
**Date**	**07-Jul-2026**
**COI**	**None**

No

Reviewer **3**

Name **Serra-Sutton, Vicky**

Affiliation **Agència de Qualitat i Avaluació Sanitàries de Catalunya (AQuAS) and CIBER d'Epidemiologia i Salut Pública (CIBERESP), Quality and Acomplishment area**

Date **24-Aug-2026**

COI **None**

Congratulations for a sound and innovative study. The protocol is clear and robust. Some minor considerations would increase future relevance and even more clarity of the process.

ABSTRACT:

-the general objective of the study should be included following a suggestion of change in how it is proposed to be change in the main manuscript to gain clarity and independence.

-add sample size expected in each round of consensus.

-detail how the literature review and qualitative study before the Delphi will be considered (formulation of statements, domains, discussion of findings).

-reference the platform and mention that it will be an online synchronic or a synchronic Delphi

CONFLICTS OF INTEREST:

Authors could explicit conflicts of interest, and interests behind the present study. In this sense, they seem to promote full-time employment against part-time employment. Which strategies will allow to increase reflexivity and independence of formulation of statements, analysis, results description and discussion?

INTRODUCTION:

-When the goal states that the study's purpose is to achieve consensus, this should be changed to describe the level of consensus among selected experts. In this sense, it is important for transparency describe in which statements reach the pre-defined level of consensus and in which null, low, moderate or high degrees of consensus were reached.

-Some key information described in Discussion section could be moved to Introduction for clarity for example 2nd paragraph on page 9. The evidence of negative / positive effects of part-time employment versus full -time positions for organizations, management, clinical, professionals, patient/ families/ systems would need to be stated at the beginning to understand the rationale of the Delphi.

-In this sense, if limited robust evidence of benefits, a consensus study can allow to increase knowledge of these benefits, impacts barriers or facilitators of a specific topic or innovation. Perhaps state this as a strength of the present study.

-When mentioning unwanted part-time employment on page 3 "Background and rationale". Also, more explicit profiles of healthcare professionals. Are they medical doctors? Aren't nurses in this category?

-When the goal and research question are described mention to patient-facing care staff working on rotating shift [...] is introduced without completely contextualizing this aspect in Introduction. This should be further explained or contextualized at the begging. Move relevant information to Introduction.

METHODS:

-A little more detail of how evidence from the literature and qualitative study before the Delphi should be added, also in Figure 1. As example of application of evidence from literature before a Delphi study please find the following reference is useful: https://pubmed.ncbi.nlm.nih.gov/37267891/

-In this sense, it would be important to explicit how domains /section will be proposed in the different rounds of consensus.

-Will participants in the previous qualitative study participate in the Delphi? No mention to pilot testing is included and would be important to describe, together with a little more detail of inclusion/ exclusion criteria.

-The sample size is justified. Nevertheless, it limits subgroup analysis according to the profiles described

-Representatives of patients, users, family associations seem not to be included. Which is the reason and please detail more in limitations if not participating.

-When describing data analysis on page 8, “social prescribing” seems a typo.

DISCUSSION:

-The following sentence in the second paragraph of discussion is ambitious and perhaps not accomplished in a direct way:

“The present Delphi study is expected to contribute to addressing these challenges by developing a shared conceptual understanding of full-time culture and identifying the key factors that facilitate or hinder its realisation in Norwegian municipal healthcare.”

Mention to knowledge sharing and specifically aid in defining strategies where barriers are found according to different experts profiles. Detecting future research gaps could also be relevant.

-For readers from different health Systems, a little more description of the Organization of Norwegian municipal health care could be included (primary health care and social services?). Are they differences among regions?

CONCLUSION:

- The sentence “Such strategies have the potential to mitigate workforce shortages, enhance the continuity, quality, and safety of care, and ensure that sufficient qualified personnel are available to meet the growing demand for healthcare services.” goes far beyond potential findings of the present Delphi study. This sentence should be removed. It could be further referenced or added in Discussion or Introduction section. In this sense, benefits from the consensus would allow to gain clarity on them and compare with existing evidence available that authors mention here.

VERSION 1 - AUTHOR RESPONSE

	Reviewer comments	Changes in the manuscript
	**Reviewer #1**	
1.	Comments to the Author:<br>Thank you for the opportunity to review your manuscript. The topic is relevant and timely; however, several areas require further clarification and strengthening to enhance the rigor and clarity of the paper.	We sincerely thank the reviewer for the thorough and constructive review of our manuscript. We highly appreciate the valuable feedback provided, and we have carefully addressed all comments and suggestions, which we believe have strengthened the clarity and overall quality of the manuscript.

2.	First, in the section addressing the background and necessity of the study, the description of healthcare workforce data in Norway is currently too brief. I recommend expanding this part by incorporating recent statistics related to the "full-time work culture" among healthcare professionals in Norway. In particular, please clarify the extent to which full-time positions are lacking, why such shortages persist, and what structural or organizational factors contribute to this issue. Additionally, it would be important to explicitly discuss the consequences of this shortage, such as its impact on continuity of care, staff well-being, quality of services, and organizational efficiency. Providing this context will help readers better understand the significance and urgency of the research problem.	We agree that a more comprehensive description of part-time employment and the challenges associated with developing a full-time work culture in Norwegian healthcare is important for understanding the background and relevance of the study.<br><br>We have therefore expanded the background section to provide additional context on the extent and persistence of part-time employment in Norwegian healthcare. We have added recent evidence showing that approximately one in four part-time employees report that they would prefer a full-time position [Nielsen et al., 2026]. We added a structural explanation of the persistence of part-time employment, highlighting how working-time reforms introduced in 1987, including the transition from working every second weekend to every third weekend, increased staffing requirements and contributed to greater reliance on part-time positions to ensure adequate weekend coverage [Moland, 2013].<br><br>In addition, we have expanded the discussion of the consequences of part-time employment.
3.	Second, the purpose of the study would benefit from clearer alignment with the identified gaps. Specifically, please articulate more explicitly how the lack of a full-time work culture and related workforce challenges motivated the present study. Making this connection more explicit will improve the logical flow of the manuscript and help readers better appreciate the contribution of your research.	To address this concern, we have expanded the background section by adding evidence on the consequences of part-time employment for employees, care delivery, and workforce sustainability. The revised text now emphasises that increasing employment fractions may strengthen workforce capacity, employee engagement, professional development, and continuity of care. This addition provides a clearer rationale for the study and more explicitly links the identified gap regarding the lack of a full-time work culture to the workforce challenges facing health and care services, thereby clarifying the motivation for the present research.
4.	Finally, in the Discussion section, some of the cited references appear outdated. For example, references [17] (1992) and [18] (2014) are more than a decade old. Given the rapid changes in healthcare systems and organizational culture, these sources may not	We have removed the older references and incorporated a recent review on organisational culture in healthcare (Efremov et al., 2025) to strengthen the currency and relevance of the discussion.

	adequately reflect current hospital work environments. I strongly recommend replacing or supplementing these references with more recent literature that captures contemporary<br>organizational culture in healthcare settings. This will strengthen the validity and relevance of your discussion.	
	**Reviewer #2**	
1.	Reviewer: 2<br>Comments to the Author:<br>No	We thank Reviewer 2 for reviewing our manuscript and for indicating that no revisions were required.
	**Reviewer #3**	
1.	Comments to the Author:<br>Congratulations for a sound and innovative study. The protocol is clear and robust. Some minor considerations would increase future relevance and even more clarity of the process.	Thank you for the positive evaluation of our study and for the valuable suggestions. We tried to address the comments in the revised manuscript.
2.	ABSTRACT:<br>-the general objective of the study should be included following a suggestion of change in how it is proposed to be change in the main manuscript to gain clarity and independence.<br>-add sample size expected in each round of consensus.<br>-detail how the literature review and qualitative study before the Delphi will be considered (formulation of statements, domains, discussion of findings).<br>-reference the platform and mention that it will be an online synchronic or a synchronic Delphi	We have revised the abstract to improve its clarity and alignment with the revised study aim and methodology.<br>First, we have explicitly stated the general aim of the study. Second, we have added the expected panel size in various rounds (n=20), subject to participant retention. Third, we have clarified the process by which the Delphi content will be developed. The Delphi will begin with an open-ended qualitative Round 1. Responses will be analysed thematically and synthesised into domains and statements, which will form the basis for Round 2. Therefore, the statements used in the subsequent consensus rounds will be generated from the participants' responses rather than being predefined. Finally, we have specified that the study is a three-round, asynchronous online Delphi conducted using the Welphi platform.
3.	CONFLICTS OF INTEREST:<br>Authors could explicit conflicts of interest, and interests behind the present study. In this sense, they seem to promote full-time employment against part-time employment. Which strategies will allow to increase reflexivity and independence of formulation of statements, analysis, results description and discussion?	We agree that the study should clearly distinguish between exploring a full-time work culture and advocating for full-time employment. To address this concern, we have added statements in both the Strengths and Limitations and Discussion sections clarifying that the aim of the study is not to promote full-time employment per se, but to explore expert perspectives on opportunities, barriers, and

		potential consequences related to a full-time work culture. We also emphasise that both full-time and part-time employment may have advantages and disadvantages. Furthermore, we have expanded the Introduction by incorporating research into the potential consequences of part-time employment and the potential benefits of increased employment fractions for employees, organisations, and quality of care.
4.	INTRODUCTION:<br>When the goal states that the study's purpose is to achieve consensus, this should be changed to describe the level of consensus among selected experts. In this sense, it is important for transparency describe in which statements reach the pre-defined level of consensus and in which null, low, moderate or high degrees of consensus were reached.	The final section of introduction was improved to remove unclarity as follows: ... this study uses a Delphi technique to assess the level of consensus among a panel of experts regarding the definition, facilitators, and key barriers to developing and sustaining a full-time culture in Norwegian healthcare. Also, it is going to identify which statements reach the predefined level of consensus and to describe the degree of agreement across statements that do not reach this threshold.
5.	-Some key information described in Discussion section could be moved to Introduction for clarity for example 2nd paragraph on page 9. The evidence of negative / positive effects of part-time employment versus full -time positions for organizations, management, clinical, professionals, patient/ families/ systems would need to be stated at the beginning to understand the rationale of the Delphi.	We have moved relevant text from the Discussion section to the Introduction and expanded the background section accordingly. The Introduction now includes evidence on the potential implications of part-time employment for employee engagement, professional development, continuity of care, and workforce sustainability, while also acknowledging that findings are mixed in some areas. We believe these additions provide a clearer overview of the potential positive and negative effects of part-time employment and strengthen the rationale for conducting the Delphi study.
6.	-In this sense, if limited robust evidence of benefits, a consensus study can allow to increase knowledge of these benefits, impacts barriers or facilitators of a specific topic or innovation. Perhaps state this as a strength of the present study.	Thank you for this valuable suggestion. We have added a strength highlighting that the Delphi approach provides consensus-based knowledge about the potential benefits of full-time culture in a field where empirical evidence remains limited.
7.	-When mentioning unwanted part-time employment on page 3 “Background and rationale”. Also, more explicit profiles of healthcare professionals. Are they medical doctors? Aren't nurses in this category?<br>-When the goal and research question are described mention to patient-facing care staff working on rotating shift [...] is introduced	We have revised the Introduction to clarify who this group comprises, namely healthcare assistants, auxiliary nurses, practical nurses, and registered nurses providing direct patient care in municipal health and care services. We also clarify that physicians are generally not affected by involuntary part-time employment, as part-time work is uncommon in this professional

	without completely contextualizing this aspect in Introduction. This should be further explained or contextualized at the begging.<br><br>Move relevant information to Introduction.	group, and are therefore outside the scope of this study. In addition, we expanded the background section to explain why involuntary part-time employment is particularly relevant among shift-working care staff and how historical working-time arrangements in Norwegian healthcare have contributed to the widespread use of part-time positions. These additions provide a clearer rationale for focusing on patient-facing care staff working rotating shifts and establishing the context for the study aim and research questions.
8.	METHODS:<br>A little more detail of how evidence from the literature and qualitative study before the Delphi should be added, also in Figure 1. As example of application of evidence from literature before a Delphi study please find the following reference is useful: https://pubmed.ncbi.nlm.nih.gov/37267891/<br><br>-In this sense, it would be important to explicit how domains /section will be proposed in the different rounds of consensus.<br><br>-Will participants in the previous qualitative study participate in the Delphi? No mention to pilot testing is included and would be important to describe, together with a little more detail of inclusion/ exclusion criteria.	We have revised the Methods section and updated Figure 1 to clarify the sequential development of the Delphi study. Specifically, we now state that Round 1 consists of open-ended questions, designed to elicit experts' perspectives on full-time work culture.<br>Round 2 presents statements derived from the Round 1 responses for rating and evaluation, while subsequent rounds are used to refine and achieve consensus.<br><br>We have added information to the Methods section stating that the Welphi platform, study materials, and survey procedures will be pilot tested by five individuals (two researchers and three healthcare leaders) prior to data collection. The purpose of the pilot test is to assess clarity, usability, and technical functionality of the survey process. Pilot participants will not be included in the Delphi panel or the study analyses.<br>We have also expanded the description of the inclusion and exclusion criteria to clarify participant eligibility for the Delphi study.<br><br>The responses will be analysed and grouped into thematic domains. These domains will form the basis for the development of statements that will be presented for rating in Round 2. In subsequent rounds, participants will re-evaluate the statements and provide additional comments, allowing the domains and their associated items to be refined until consensus is achieved.
9.	-The sample size is justified. Nevertheless, it limits subgroup analysis according to the profiles described	We agree that the anticipated sample size limits the possibility of meaningful comparisons between stakeholder subgroups. We have clarified this limitation in the revised

		manuscript and specified that the Delphi study is designed to establish overall expert consensus rather than to provide statistically powered subgroup analyses. Participant characteristics will be described, and any patterns of agreement across stakeholder groups will only be explored descriptively where the sample permits, without drawing conclusions about statistically generalisable subgroup differences.
10.	-Representatives of patients, users, family associations seem not to be included. Which is the reason and please detail more in limitations if not participating.	We have clarified the inclusion and exclusion criteria in the revised manuscript. Patients, service users, and relatives were excluded because the objective of the Delphi study is to establish consensus among experts with relevant organisational, managerial, and workforce-related knowledge of full-time culture. We acknowledge that patients, service users, and family members may offer valuable perspectives on how employment practices affect care experiences and outcomes. However, the focus of this study is on organisational factors influencing the development of a full-time work culture, an area that requires specific expertise in workforce organisation and healthcare management. This rationale has now been clarified in the manuscript.
11.	-When describing data analysis on page 8, "social prescribing" seems a typo.	Thank you for identifying this error. "Social prescribing" was a typographical error and has been corrected to "full-time culture" in the revised manuscript.
12.	DISCUSSION:<br>-The following sentence in the second paragraph of discussion is ambitious and perhaps not accomplished in a direct way: "The present Delphi study is expected to contribute to addressing these challenges by developing a shared conceptual understanding of full-time culture and identifying the key factors that facilitate or hinder its realisation in Norwegian municipal healthcare."<br>Mention to knowledge sharing and specifically aid in defining strategies where barriers are found according to different experts profiles.	We agree that the original wording was too ambitious regarding what can be directly achieved through a Delphi study. We have therefore revised the paragraph to clarify that the study aims to bring together knowledge and perspectives from experts with different professional and organisational backgrounds, identify areas of consensus as well as barriers and facilitators across expert profiles, and contribute to knowledge sharing. We also clarify that the findings may inform the development of context-sensitive strategies and identify areas where targeted approaches or further dialogue may be needed. Finally, we have added that the findings may highlight gaps

	Detecting future research gaps could also be relevant.	in current knowledge and provide directions for future research.
13.	For readers from different health Systems, a little more description of the Organization of Norwegian municipal health care could be included (primary health care and social services?). Are they differences among regions?	We agree that an introductory description of the Norwegian healthcare system will provide a stronger contextual understanding for the remainder of the article. We have therefore added such a description, along with two new references in this regard.
14.	CONCLUSION:<br>- The sentence "Such strategies have the potential to mitigate workforce shortages, enhance the continuity, quality, and safety of care, and ensure that sufficient qualified personnel are available to meet the growing demand for healthcare services." goes far beyond potential findings of the present Delphi study. This sentence should be removed. It could be further referenced or added in Discussion or Introduction section. In this sense, benefits from the consensus would allow to gain clarity on them and compare with existing evidence available that authors mention here.	We have removed the standalone Conclusion section. The relevant text has been revised, made more cautious, and integrated into the final paragraph of the Discussion.
